# Knee Injury Detection Using Deep Learning on MRI Studies: A Systematic Review

**DOI:** 10.3390/diagnostics12020537

**Published:** 2022-02-19

**Authors:** Athanasios Siouras, Serafeim Moustakidis, Archontis Giannakidis, Georgios Chalatsis, Ioannis Liampas, Marianna Vlychou, Michael Hantes, Sotiris Tasoulis, Dimitrios Tsaopoulos

**Affiliations:** 1 Department of Computer Science and Biomedical Informatics, School of Science, University of Thessaly, 35131 Lamia, Greece; stas789@gmail.com; 2 Centre for Research and Technology Hellas, 38333 Volos, Greece; d.tsaopoulos@certh.gr; 3 AIDEAS OÜ, 10117 Tallinn, Estonia; s.moustakidis@aideas.eu; 4 School of Science and Technology, Nottingham Trent University, Nottingham NG11 8NS, UK; archontis.giannakidis@ntu.ac.uk; 5 Department of Orthopedic Surgery, Faculty of Medicine, University of Thessaly, 41500 Larissa, Greece; ghalatsis@hotmail.com (G.C.); hantesmi@otenet.gr (M.H.); 6 Department of Neurology, School of Medicine, University Hospital of Larissa, University of Thessaly, Mezourlo Hill, 41500 Larissa, Greece; liampasioannes@gmail.com; 7 Department of Radiology, School of Health Sciences, University Hospital of Larissa, University of Thessaly, Mezourlo, 41500 Larissa, Greece; mvlychou@uth.gr

**Keywords:** ACL, deep learning, knee injury, machine learning, meniscus

## Abstract

The improved treatment of knee injuries critically relies on having an accurate and cost-effective detection. In recent years, deep-learning-based approaches have monopolized knee injury detection in MRI studies. The aim of this paper is to present the findings of a systematic literature review of knee (anterior cruciate ligament, meniscus, and cartilage) injury detection papers using deep learning. The systematic review was carried out following the PRISMA guidelines on several databases, including PubMed, Cochrane Library, EMBASE, and Google Scholar. Appropriate metrics were chosen to interpret the results. The prediction accuracy of the deep-learning models for the identification of knee injuries ranged from 72.5–100%. Deep learning has the potential to act at par with human-level performance in decision-making tasks related to the MRI-based diagnosis of knee injuries. The limitations of the present deep-learning approaches include data imbalance, model generalizability across different centers, verification bias, lack of related classification studies with more than two classes, and ground-truth subjectivity. There are several possible avenues of further exploration of deep learning for improving MRI-based knee injury diagnosis. Explainability and lightweightness of the deployed deep-learning systems are expected to become crucial enablers for their widespread use in clinical practice.

## 1. Introduction

### 1.1. Backdrop

Knee injuries account for the largest percentage of sport-related, severe injuries (i.e., injuries that cause more than 21 days of missed sport participation) [1,2,3,4]. Anterior cruciate ligament (ACL) ruptures represent more than 50% of the cases, affecting 200,000 individuals in the United States each year [1,5,6,7]. Knee cartilage lesions affect around 900,000 individuals in the United States every year, resulting in over 200,000 surgical procedures [5,6,7,8]. Menisci injuries are the second most common knee impairment, with an incidence of 12–14% [9] and a prevalence of 60–70 cases per 100,000 in the United Kingdom [2]. ACL injuries alone account for an expenditure of more than $7 billion in the United States [10]. Both short- and long-term pain, disability, and negatively affected, health-related quality of life have all been strongly associated with knee injuries [11,12,13]. In regard to young and athletic individuals, the more time they spend engaging in occupational and/or recreational activities, the higher predisposition to knee injuries they have, which, in turn, contributes to a higher likelihood of developing osteoarthritis (OA) [14]. On average, half of the individuals, that have an injury that involved ACL and/or meniscal tear develop radiographically confirmed knee OA ten to 20 years post-injury [15,16]. Another two possible consequences of knee injuries are: (i) structural muscle injuries of the lower limb [17]; and (ii) tendinopathies [18]. All the above reflect the direct and indirect (lost wages, productivity, and disability) socio-economic burden conferred on the society by knee injuries. The high prevalence of knee injuries in the general population, and the resulting socio-economic impact, have created a necessity for developing accurate and cost-effective procedures that can detect and quantify the severity of knee injuries. Early diagnosis and, consequently, treatment of ligament rupture, menisci tear, and/or cartilage lesion can prevent early onset of knee OA [1].

Arthroscopy is considered the “gold-standard” for the diagnosis of intra-articular knee pathologies, but is limited by potential complications and its invasive nature [19]. Therefore, magnetic resonance imaging (MRI) is the most widely used, non-invasive imaging technique for diagnosing knee injuries [20,21]. However, the MRI-based diagnosis of knee injuries can be a very challenging procedure, with the experience of clinicians playing a critical role in image interpretation. Human-based image interpretation pitfalls, such as subjectivity, distraction, and fatigue, as well as diagnostic uncertainties, often lead to erratic diagnoses, hindering the optimal management of knee injuries [22,23]. Moreover, clinical-diagnostic discrepancies among non-musculoskeletal radiologists and orthopedic surgeons are commonly encountered in everyday clinical practice [11].

Due to the above-listed factors, as well as the exponentially increasing number of clinical examinations, the idea of using computers for improving the challenging task of image interpretation of medical examinations has been recently adopted by the scientific community [24]. Imaging data proliferation, algorithmic advances, and recent technological advances in fast computing have already resulted in a strong push towards the utilization of artificial intelligence (AI) algorithms in medical image analysis. The term AI broadly refers to any method that enables computers to mimic human intelligence [25]. Deep learning (DL) in particular is a class of machine-learning (ML) algorithms that is currently driving the AI boom [26]. Numerous applications of DL in medical image analysis have been reported, including skin cancer classification, diabetic retinopathy detection, lung nodule detection, and mammography cancer detection, among others [27]. The aforementioned AI-empowered solutions are expected to revolutionize medical sectors by improving the accuracy and productivity of different diagnostic and therapeutic measures in clinical practice [20].

Drawing attention to the diagnosis of knee injuries, several early DL studies have exhibited better performance than traditional ML techniques, while in some cases they have proved to be even superior to radiologists [26]. However, the previously published review studies in the MRI field were either focused on other application domains (e.g., fracture detection [28]) or limited to the performance of the proposed networks without paying attention to their specifics (learning methodology, processing stages, technical limitations etc.) [29]. In the light of the advancements of AI technology and the increasing number of studies in this field, in this paper, we conducted a systematic literature review, covering all DL-oriented techniques that have been employed in the diagnostic process of knee injuries. The aim of this systematic review is to identify all recent studies that investigate the use of DL technology in the MRI-based diagnosis of the injured knee. It was decided that the primary focus would be on studies that examine at least one of the following pathologies: (i) injuries of knee ligaments; (ii) meniscus tears; and (iii) cartilage lesion.

### 1.2. Machine Learning in a Nutshell: Definitions and Terminology

To enhance the understanding of the readers and for the sake of completeness, this section quickly presents the relevant terminology and definitions with respect to ML and DL algorithms used in the studies involved in the present review. ML is a branch of AI that focuses on the development of algorithms that automatically learn to make accurate predictions by relying on experience (data) rather than on hard-coded instructions.

**Figure 1:**
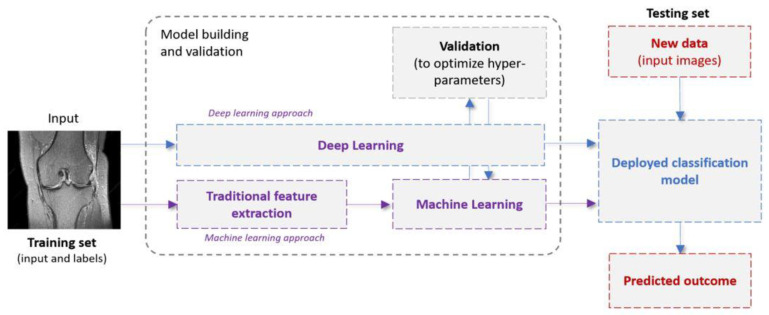
Examples of typical machine-learning and deep-learning pipelines.

Supervised ML systems (Figure 1) operate in two phases: the learning phase (training) and the testing one. In a traditional ML pipeline, a feature extraction/selection stage (also referred to as feature engineering) is first implemented to extract or identify the most informative features [16]. These features can be extracted from the input images, employing various algorithms including grey-level co-occurrence matrix (GLCM), first- and second-order statistics, and shape/edge features, among others [30]. Next, a ML model is fit to the extracted features and the optimal model parameters are obtained. During the testing phase, the trained model is shown previously unseen samples (represented as images or features extracted from images), which are then classified. As opposed to traditional programming, where the rules are manually crafted by a programmer, a supervised ML algorithm automatically formulates rules from the data.

DL [31] is a subfield of ML that sets an alternative architectural paradigm by shifting the process of extracting features from images to the underlying learning mechanism. The most informative features for the task at hand are extracted by the algorithm itself. The mainstream DL architecture for computer vision applications is the convolutional neural network (CNN). A CNN typically consists of multiple building blocks (layers such as convolutional, pooling, and fully connected) that automatically extract increasingly abstract spatial hierarchies of features. The CNN training is carried out via a backpropagation algorithm. The huge popularity of CNNs is attributed to certain characteristics they possess, such as weight sharing and spatial invariance.

Transfer learning is a common strategy where a network, that was pre-trained on a big dataset, is partly re-used to provide decisions on a problem with a different dataset. The main idea behind transfer learning is that generic features learned on a large dataset could be useful and applicable to other domain tasks with a potentially limited amount of accessible data. Numerous pre-trained networks are currently available, such as DenseNet [32], AlexNet [33], and VGG [34]. When employing DL with transfer learning for feature extraction, the pre-trained network is treated as an arbitrary feature extractor: the input image propagates through multiple layers until it reaches a pre-specified layer, the outputs of which are considered as the finally extracted features. Table 1 provides a brief presentation of the main ML and DL algorithms that were reported in the papers of this review.

## 2. Materials and Methods

### 2.1. Reporting

The present (systematic) review was performed in accordance with the preferred reporting items for systematic reviews and meta-analyses (PRISMA) guidelines [46]. Each step of the review process (literature search, study selection, and data extraction) was independently performed by 2 authors (A.S., S.M.). Discrepancies were resolved by a 3rd author (D.T.). The present study was not registered in a database prior to its conduction.

### 2.2. Literature Search

A structured literature search was conducted in the following databases: (a) MEDLINE (through PubMed), (b) CENTRAL (through Cochrane Library), and (c) EMBASE (through Elsevier). Articles cited by the retrieved papers, as well as articles citing the retrieved papers (using Google Scholar), were also identified through a supplementary manual search. Grey literature was examined based on conference abstracts, English abstracts (from articles not published in English), and the OpenGrey database. The potential eligibility of the articles was initially decided based on their title and abstract. Full texts were investigated to verify whether the initial qualifiers fulfill the inclusion criteria. The structured search strategy per database is quoted in Table S1.

### 2.3. Eligibility Criteria

The inclusion criteria were as follows: (i) papers were published between the 1st of January 2013 (the dawn of the DL era) and the 15th of November 2021 (date of final literature search); (ii) MRI images were used for the evaluation of knee injuries; (iii) knee-injury detection was conducted via AI-based algorithms, including both traditional ML and DL techniques; and (iv) the performance of the AI-based algorithms was compared to clinical, human-based evaluations.

Papers were excluded according to the following criteria: (i) articles published before 2013; (ii) papers investigating OA or other bone pathology not directly linked with knee injuries; (iii) studies performed in animals; (iv) non-original research articles, such as protocols, reviews, meta-analysis, etc.; (v) articles not written in English (however, English abstracts were assessed as part of the grey literature); and (vi) book chapters, editorials and commentaries.

### 2.4. Data Extraction

Extracted data were placed into a custom Microsoft Excel spreadsheet using a standardized table. The following information was included for each of the articles: first author, publication year, database, description of data and models, and learning algorithm, including pre-processing, size of training and test samples, validation method, and obtained results.

### 2.5. Statistical Analysis

Multiple evaluation criteria were employed to assess the predictive capacity of the proposed learning algorithms. The most common evaluation metric considered in this review study was accuracy, along with the receiver operator characteristic curves (ROCs) that visualize the performance of a classification model at various likelihood ratio thresholds. These curves plot two factors: true positive rate (sensitivity = TP/(TP + FN)) versus the false positive rate (specificity = FP/(FP + TN)), where TP, FN, FP, and TN denote true positives, false negatives, false positives, and true negatives, respectively. The quantitative output of this curve is the AUC, which could be interpreted as an aggregated measure of performance across all possible classification thresholds.

### 2.6. Quality Assessment

Quality assessment was performed using a modified methodologic index for nonrandomized studies (MINORS) [47]. A seven-item checklist was considered, including information with respect to the following items: disclosure, study aim, input feature, ground truth label determination, dataset distribution, performance metric, and explanation of the applied AI models. Data were extracted and recorded using standardized forms (Microsoft Excel spreadsheet). To resolve conflicts over article selection, quality assessment, and data extraction, both observers (A.S., S.M.) convened a consensus meeting. The items were scored with 0 (not reported), 1 (reported but inadequate), or 2 (reported and adequate). The average modified MINORS score among all studies was 9.82 ± 1.99. It should be mentioned that the range of the score per item was between 0 and 44.

**Figure 2:**
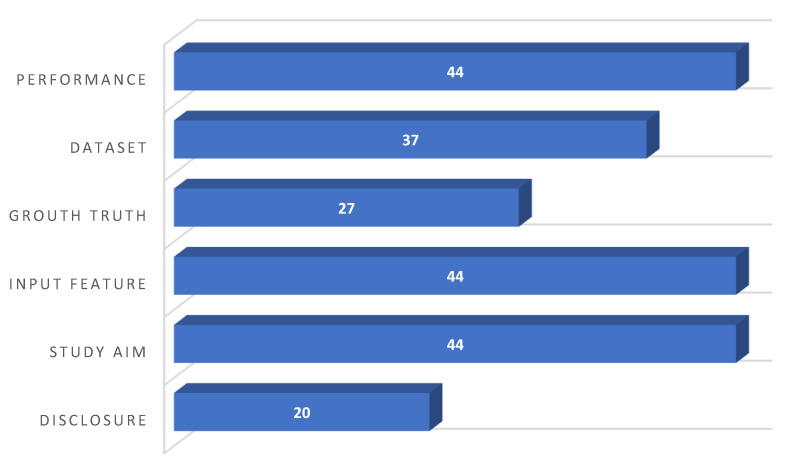
Quality assessment outcomes using the MINORS tool.

As shown in Figure 2, all the reported studies (22) clearly stated the study aim, input features, and the performance achieved using appropriate metrics. A clear distribution and description of the dataset were reported in twenty studies (90.09%). Fifteen studies (68.18%) clearly described how they established the ground truth (AI’s reference standards), whereas the others were subjected to AI models that were inadequately trained. The most prevalent causes of quality point loss were failures to describe ground truth assignment. Last, but not least, more than half of the studies (54.54%) failed to disclose a conflict of interest declaration.

## 3. Results

**Figure 3:**
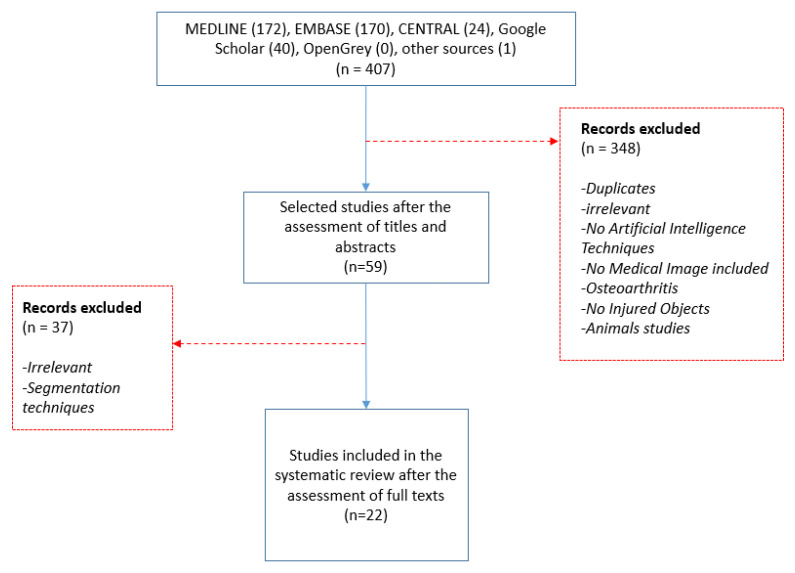
Flow chart presenting the design of the literature search.

In total, 407 studies were retrieved: 172 from MEDLINE (through PubMed), 170 from EMBASE (through ELSEVIER), 24 from CENTRAL, 40 from the structured search in Google Scholar, and 1 conference abstract (grey literature). Fifty-nine papers were selected after applying the proposed inclusion/exclusion criteria. Thirty-seven studies were further excluded due to irrelevant content (for example, those focusing only on segmentation or other scientific fields). Taking everything into consideration, 22 articles were finally included in the present systematic review. A flow chart of the literature search design is presented at Figure 3.

The retrieved articles were categorized into the following application domains: (i) detection of ACL injuries alone (10 studies); (ii) detection of meniscus tears alone (7 studies); (iii) detection of cartilage lesions (1 study); and (iv) combined ACL and meniscus tears plus other knee injuries (4 studies). The main results of each study have been quoted, while the individual study validity has been determined based on its methodological strengths and weaknesses. Important methodological features of the retrieved articles have been commented.

The identified studies focusing on ACL and meniscus tears detection have been grouped into three categories: (i) those employing traditional ML pipelines; (ii) DL studies in which transfer learning is reported; and (iii) papers that propose the use of custom-made DL architectures.

### 3.1. ACL Injury Detection

#### 3.1.1. Machine Learning

Štajduhar and colleagues [48] utilized two different feature extraction techniques: histogram oriented gradient (HOG) [35] and generalized search tree (GIST) [30]. These feature extraction techniques were subsequently paired with two commonly used ML models: support vector machines (SVMs) [38] and random forests [49]. They found that the best performing ML model was the one that combined HOG with linear-kernel SVM, producing an AUC of 0.89 for differentiating between ACL-injured and healthy subjects, and an area under curve (AUC) of 0.94 for detecting only completely ruptured ACL. Abdulah et al. [50] described a diagnostic system consisting of image pre-processing, feature extraction based on segment-derived spatial descriptors (perimeter, area, and shape), and, finally, classification. They compared k-nearest neighbor (K-NN) with back propagation artificial neural network (BP-ANN) for ACL tear classification. BP-ANN achieved a classification accuracy of 94.44% whereas K-NN reached an accuracy of 87.33%. Another study [51] tested an SVM algorithm on a dataset that was comprised of 100 non-injured ACLs, 100 partially-torn ACLs, and 100 completely-torn ACLs. All datasets underwent pre-processing. Features were extracted using shape descriptors, such as objects’ contour circularity, aspect ratio, angle, and number of sides. It was reported that the SVM model had an accuracy of 100% for classifying ACL MRI samples as normal, partial-tear, or complete-tear. The authors also sought to compare the diagnostic capability of their AI model with that of two medical experts on a subset of 10 samples. No statistically significant differences between the AI model and the radiologists were found.

#### 3.1.2. Deep Learning with Transfer Learning

Bien et al. [27] used transfer learning in order to train a fully automated CNN for classifying MRI series and they combined the predictions from 3 series per exam using logistic regression. The accuracy and the AUC of the model for detecting ACL tears were 86.7% and 0.965, respectively. These results were juxtaposed with the assessments by three musculoskeletal (MSK) radiologists on a testing set of 120 knee MR images. Radiologists achieved significantly higher sensitivities for tear diagnosis than the AI model (AUC: 0.91 vs. 0.76, *p*-value = 0.002). The accuracy achieved by the radiologists (92%) was higher than the one achieved by the AI model (86.7%). Azcona et al. [52] proposed and evaluated the performance of four architectures: (i) deep residual network with transfer learning; (ii) custom deep residual network using a fixed number of slices; (iii) multi-plane deep residual network; and (iv) multi-plane multi-objective deep residual network. They found that transfer learning combined with a carefully tuned data augmentation strategy were the crucial factors in achieving best performance. The authors modified the last layer to output a probability instead of a one-hot softmax vector for a number of classes and they also used transfer learning with pre-trained weights from ImageNet. By using the aforementioned DL architectures and data augmentation strategies for ACL detection, they achieved an AUC of 0.96 on the validation data.

#### 3.1.3. Custom-Made Deep-Learning Networks

Another study [8] evaluated three customized CNN models with variations in the input fields of view (i.e., full slice, cropped slice, and dynamic patch-based sampling) as well as in dimensionality (single slice, three slices, or five slices) for the detection of complete ACL tears. The importance of limiting the input field-of-view to the intercondylar region for high algorithm performance was demonstrated. The incremental value of contextual information of adjacent image slices in improving network classification accuracy was also exhibited. The model that utilized dynamic sampling had an accuracy of 96.7% and an AUC of 0.97. Liu et al. [53] trained multiple CNNs and applied them to a test set comprised of 50 MR images of ACL tears with normal thickness and 50 MR images with intact ACLs. The best model they came up with for detecting the presence or absence of a full thickness ACL tear produced an AUC of 0.98 (95% CI: 0.93–1.00, *p*-value < 0.001). However, there was no statistically significant difference in diagnostic performance between the AI model (AUC: 0.98, 95% CI: 0.93- 1.00) and the clinical radiologist performance: Radiologist AUC: 0.98 (95% CI: 0.95–1.00); Fellow AUC: 0.98 (95% CI: 0.95- 1.00); Resident 1 AUC: 0.93 (95% CI: 0.88–0.98); Resident 2 0.97 (95% CI: 0.94–1.00); Resident 3 0.98 (95% CI: 0.95–1.00).

Namiri et al. [54] employed two CNN types for classification of ACL injuries: the first one involved three-dimensional (3D) kernels, whereas the second one made use of two-dimensional (2D) filters. The overall accuracies using the 3D CNN and the 2D CNN were 89% (225 of 254) and 92% (233 of 254), respectively (*p*-value= 0.27), whereas both CNNs had a weighted Cohen k of 0.83. The 2D CNN and 3D CNN performed similarly in classifying intact ACLs (2D CNN: sensitivity of 93% and specificity of 90%; 3D CNN: sensitivity of 89% and specificity of 88%). The classification of full tears by both networks was also comparable (2D CNN: sensitivity of 82% and specificity of 94%; 3D CNN: sensitivity of 76% and specificity of 100%). The 2D CNN classified all reconstructed ACLs correctly. A separate study [6] proposed to perform CNN-based classification by relying on the architecture of 3D DenseNet [32]. They compared this DL approach with two other variants, namely VGG16 [34] and ResNet [42]. The accuracy, sensitivity, specificity, positive predictive value (PPV), and negative predictive value (NPV) of the proposed customized architecture were calculated respectively. The average AUCs were 0.95, 0.86, and 0.96 for ResNet, VGG16, and their proposed network, respectively. The diagnostic accuracies achieved by the proposed model, the residents, and the senior radiologists were 95.7%, 81.4%, and 89.9%, respectively.

Germann et al. [24] trained a deep convolutional neural network (DCNN) on 512 MR images of ACL tears from different patients (ACL tears were present in 45.7% and absent in 54.3% of the subjects). The network had a pre-processing step that involved the selection, rescaling, and cropping of coronal and sagittal-fluid-sensitive views. Next, the coronal and sagittal MRI scans were processed independently in parallel and were then concatenated before being processed by one dense layer fat-suppressed MRI scan. Finally, a soft-max layer extracted the confidence level for the ACL tear. Three fellowship-trained full-time academic MSK radiologists independently evaluated the MRI examinations for full-thickness ACL tears. ACL tears were present in 45.7% and absent in 54.3% of the subjects. The DCNN had a sensitivity of 96.1%, which was not significantly different from that of the readers (97.5–97.9%; all *p*-values ≥ 0.118). However, the sensitivity of the DCNN (93.1%) was significantly lower than that of the readers (99.6–100%, all *p*-values < 0.001), and a similar trend was observed in the AUC values (DCCN: 0.94, readers: 0.99–0.99, all *p* < 0.001). Finally, a related study [55] used a customized 14-layer ResNet-based CNN with six different directions by using class balancing and data augmentation. The proposed ResNet-14 achieved AUC values of 0.98, 0.97, and 0.99 for detecting a healthy tear, partial tear, and fully ruptured tear, respectively. Jeon et al. [56] proposed a 3D deep-neural-network model for diagnosing ACL tears from a knee MRI test that is both interpretable and lightweight. They used squeeze modules and fewer convolutional filters to represent the homogeneity of the features, as well as attention modules and Gaussian positional encoding to strengthen the searching of local features. Their model outperformed the prior SOTA on the Chiba and Stanford knee datasets, achieving average ROC and AUC values of 0.983 and 0.980, respectively. Recently, Dai et al. introduced TransMed [57] as a multi-modal medical picture categorization system. It combines the benefits of CNN and transformer to efficiently extract low-level characteristics from pictures and construct long-range relationships between modalities. The accuracy and the AUC of the model for detecting ACL tears were 94.9% and 0.98, respectively. These results were of higher accuracy than the MRNet technique. Astuto et al. [58] made use of 3D CNNs, which were designed to identify and grade ACL injuries in MRI investigations. The reported binary lesion sensitivity for ACL tissue is 88%. The specificity of the results is 89%. The AUC is 0.90.

### 3.2. Meniscus Tear

#### 3.2.1. Machine Learning

Fu et al. [59] compared the performance of two SVM models in detecting meniscus tears. One model was trained on selected MR features (from a pool of 180 spatial and textural features using GLCM), while the other model implemented the SVM model without any feature selection. The SVM model without feature selection produced an AUC of 0.73, while their model with feature selection yielded an AUC value of 0.91. Zarandi et al. [60] performed MR image segmentation, followed by the application of a perceptron neural network (PNN) for classifying meniscal tears. The model accomplished a 90% classification accuracy (meniscus tear versus no meniscus tear) on a testing dataset of 50 MRI studies. Precision (%) was also reported for five different settings of meniscus tear, including: (1) medial anterior horn and posterior horn normal (88.82%); (2) lateral anterior horn and posterior horn normal (92.13%); (3) medial anterior horn normal and posterior horn torn (84.24%); (4) lateral anterior horn normal and posterior horn torn (91.96%); and (5) lateral anterior horn torn and posterior horn normal (87.64%).

#### 3.2.2. Deep Learning with Transfer Learning

Another group [27] utilized MRNet as the primary building block of their prediction system, that is CNN mapping a 3D MRI series to a probability. The input to MRNet had dimensions: s × 3 × 256 × 256, where s was the number of images in the MRI series (3 is the number of color channels). In diagnosing a meniscus tear, this group reported an accuracy of 72.5% (95% CI: 0.639–0.797) and an AUC of 0.85 (95% CI: 0.78–0.91). Furthermore, they compared the performance of the proposed model with unassisted MSK radiologists for detecting a meniscus tear (intact, degenerative changes without tear, or postsurgical changes without tear). When compared to the MSK radiologists in the study, the AI model had a statistically significant lower specificity (AUC: 0.88, 95% CI: 0.85–0.91 versus AUC: 0.741, 95% CI: 0.62–0.84; *p*-value = 0.003) and accuracy (0.85, 95% CI: 0.82–0.87 versus 0.725, 95% CI: 0.64–0.80, *p*-value = 0.015). The sensitivity was also shown to be lower for the AI model (0.82, 95% CI: 0.78–0.85) compared to MSK radiologists (0.71, 95% CI: 0.59–0.81; *p*-value = 0.504), although this was not statistically significant. Azcona and colleagues [52] leveraged the baseline MRNet architecture and replaced the AlexNet feature extractor with more modern residual architectures, such as Resnet18, Resnet50, and Resnet152. They applied a series of transformations including horizontal flips and photometric augmentations (with respect to random contrast, gamma, and brightness). They reported an AUC performance of 0.91 on the validation data by using ResNet18.

#### 3.2.3. Custom-Made Deep-Learning Networks

Couteaux et al. [61] used a region-based convolutional neural network (R-CNN) model for tear detection and localization (anterior or posterior). The anterior meniscus was classified as torn when at least one network had detected a torn anterior meniscus and the posterior meniscus was classified as torn when the strict majority of the networks had detected a torn posterior meniscus. A weighted AUC score of 0.91 was achieved by the proposed network on a test set of 700 MRIs. Another paper [62] also used an R-CNN trained on a dataset of 700 MRI images to perform three tasks, namely the detection of meniscus tear presence, position, and orientation. Their AI model produced an AUC of 0.94 on the task of detecting the presence of a meniscal tear, 0.92 for detecting the position of the two meniscal horns, and 0.83 for detecting the orientation of the tear. The overall combined AUC was 0.90.

Another group [63] created a DL model that combined meniscus segmentation and a 3D CNN for accomplishing both the detection and severity staging of meniscus lesions. The segmentation task for both cartilage and the meniscus was implemented using 2D U-Net [64]. The model was first built to recognize the presence of a lesion (including intrasubstance abnormalities), and, subsequently, to quantify the lesion severity. This model produced a lesion detection AUC performance of 0.89 on the test dataset and accuracies of 80.74%, 78.02%, and 75.00% for determining severe, mild-moderate, and no lesions, respectively. Comparisons were made between the model and experts. The authors also sought to determine the inter-rater variability between three MSK radiologists (expert 1: >20 years of experience, expert 2: 10 years of experience, and expert 3: <1 year of experience) for assessing meniscus lesion severity on selected cases. They restored an average agreement among the three experts of 86.27% for no meniscus lesions, 66.48% for mild-moderate lesions, and 74.66% for severe lesions, while the best model obtained accuracies of 80.74% for no meniscus lesions, 78.02% for mild-moderate lesions, and 75.00% for severe lesions.

Fritz et al. [15] proposed that deep CNN-based meniscus tear detection be performed in a fully automated manner with a similar specificity, but a lower sensitivity, in comparison with the MSK radiologists. The AUC of the deep CNN employed was 0.88, 0.78, and 0.96 for the detection of medial, lateral, and overall meniscus tear, respectively. The sensitivity, specificity, and accuracy for medial meniscus tear detection were 93%, 91%, and 92%, respectively, for reader 1; 96%, 86%, and 92%, respectively, for reader 2; and 84%, 88%, and 86%, respectively, for the DCNN. The sensitivity, specificity, and accuracy for lateral meniscus tear detection were 71%, 95%, and 89%, respectively, for reader 1; 67%, 99%, and 91%, respectively, for reader 2; and 58%, 92%, and 84%, respectively, for the DCNN. The sensitivity for medial meniscus tears was significantly different between reader 2 and the DCNN (*p*-value = 0.039), but no significant differences were witnessed in all other comparisons (all *p*-value ≥ 0.092). Rizk et al. [65] used a 3D CNN architecture that incorporated meniscal localization and lesion classification. They achieved AUC values of 0.93 and 0.84 for medial and lateral meniscal tear detection, respectively, and 0.91 and 0.95 for medial and lateral meniscal tear migration detection, respectively. The combined medial and lateral meniscal tear detection models were externally validated and yielded an AUC of 0.83 without additional training and 0.89 after fine-tuning. Moreover, Dai et al. utilized TransMed [57], achieving accuracy and AUC values of 94.9% and 0.98, respectively, for detecting meniscus tears, thus improving over the MRNet technique. 3D CNNs were built by Astuto et al. [58] to identify and grade meniscus tear in MRI examinations. The reported binary lesion sensitivity and specificity values were 85% for both., whereas the AUC was 0.93. Lastly, Dai et al. used TransMed to also identify meniscus tears in the MRNet dataset. The group reported an AUC of 0.95 and an accuracy of 85.3%.

### 3.3. Cartilage Lesion and Other Abnormalities

Liu et al. [66] developed a fully automated DL-based cartilage lesion detection system by combining CNN-based semantic segmentation and disease classification. Segmentation was implemented via the use of a VGG-16-based encoder network consisting of a combination of 2D convolution layers, rectified-linear activations, batch normalization layers, and max-pooling layers to achieve image feature extraction and data compression at the same time. The classification CNN in the proposed pipeline was also based on the 2D VGG16. Their pipeline achieved an AUC in the range of 0.91–0.92, indicating high overall diagnostic accuracy for detecting cartilage lesions. In addition, there was good intra-observer agreement between two individual evaluations, with a k-statistic of 0.76. As previously indicated, Astuto et al. [58] also used 3D CNNs to detect cartilage lesions. The sensitivity and specificity of binary lesions were found to be 85% and 89%, respectively, whereas the AUC was 0.93. Finally, three of the reported papers [27,52,57] attempted to detect other knee abnormalities, such as osteoarthritis, effusion, iliotibial band syndrome, posterior cruciate ligament tear, fracture, contusion, plica, and medial collateral ligament sprain. MRNet [27], ResNet18 [67], and TransMed networks were employed to implement the classification tasks, achieving AUC values of 0.94, 0.94, and 0.976, respectively.

## 4. Discussion and Conclusions

**Figure 4:**
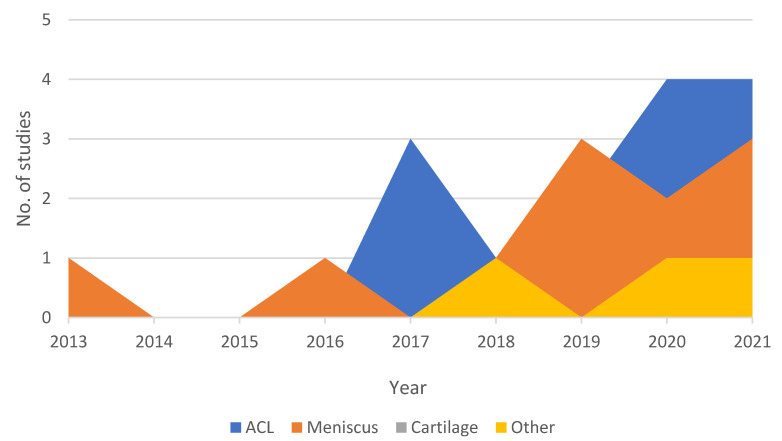
Temporal evolution chart depicting the number of ML papers per category published each year since 2013.

The present systematic review (Table 2) outlined the recent application of traditional ML and DL models to the diagnosis of the most common knee injuries using MRI as the main data source. The results of the present study can be summarized as follows. Figure 4 shows an increasing trend in adopting ML-based studies in this application area, with most of the papers being published from 2017 onwards (whilst the first ML-based paper on the field was published in 2013). Medical imaging, and specifically MRI, has to be seen as one of the most instructive assets in the field of knee injury diagnosis. The proliferation of MRI data has facilitated the effective training of ML and DL networks towards the development of: (i) novel methodologies that could enhance the medical experts’ domain knowledge and understanding of MRI; and (ii) new, data-driven tools that could enable a more reliable, fast, and fully automated detection of knee injuries. The main characteristics of the proposed MRI-based learning algorithms and pipelines were identified along with the data sources investigated. The following paragraphs present our findings with respect to the choice of CNN networks and the associated results in comparison with clinical assessments carried out by experts.

**Figure 5:**
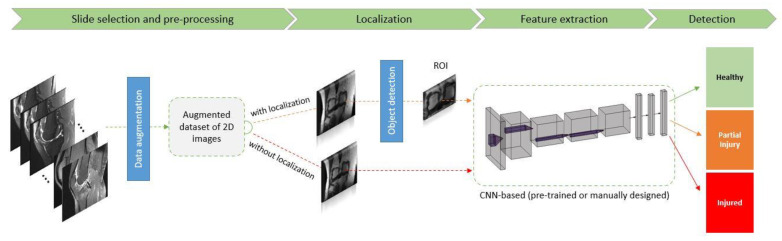
A typical DL pipeline for ACL detection.

Although there is no clear acceptance of a “gold-standard” methodological pipeline for diagnosing knee abnormalities using MRI data, it was observed that a number of processing steps were commonly employed in the majority of the reported studies. Figure 5 visualizes a DL pipeline that was adopted by most of the papers, including a pre-processing step, localization (optionally) by identifying regions of interest, and, finally, a CNN-based classification step. Data augmentation was employed by a significant number of papers in the detection of ACL injuries [6,27,52,54,55,56,57,58], in papers where meniscus injuries were investigated [27,52,57,58,62,63], and, finally, in studies focusing on cartilage lesion abnormalities [27,52]. In particular, the available MRI images were modified (via a number of image transformations such as random rotations, shifting, flipping, and the addition of noise) to expand the training dataset, and thus help to improve the performance and ability of the employed DL models to generalize. Localization was employed in papers from all three subcategories: (i) ACL studies [6,8,24,48,53,54,55,56,58]; (ii) meniscus injuries detection studies [15,58,59,60,61,62,63,65]; and (iii) for diagnosing lesion abnormalities [66]. Segmentation or objection detection algorithms were applied in the aforementioned studies to extract areas of interest, enabling the application of CNN-based models on focused and more relevant parts of the initially available images. Given that the region of interest (ROI) may appear in slightly different positions within an image and may have different aspect ratios or sizes, identifying ROIs with an automatic manner has been proven to be a crucial processing step.

CNN-inspired networks were identified as the dominant approach in the task of extracting informative features from either ROIs or entire MRIs and finally classifying them as normal (healthy) or abnormal (indicating either partial or complete tears). Transfer learning was preferred in most of the cases, allowing the training of big and powerful deep architectures, even if the amount of available data was limited. As networks require a lot of information to be trained from scratch, this technique essentially ‘steals’ knowledge from already pre-trained large networks. Specifically, ResNet variants were used in five papers [6,8,52,55,61] of this review, whereas VGG [34], AlexNet [33], and MRNet [27] were used three times [6,27,52,53,62,66]. Other pre-trained networks that were used at least once in this survey are: DenseNet [32], Le-Net [68], ImageNet [33], and R-CNN [41]. In five [48,50,51,59,60] out of the 22 studies of the present survey, more traditional ML pipelines were applied, including a separate feature engineering step (where features were manually extracted from images). SVM classification was the preferred classifier in most of the cases.

Despite the excellent capability of CNNs to come up with valuable image representations, these models lack the capacity for capturing long-range relationships. To deal with this limitation, recent research studies [44,69] have proposed employing Transformer-based architectures for various image recognition tasks. The Transformer [70] is a neural network architecture that relies on global self-attention mechanisms, and it was initially designed for sequence-to-sequence prediction. Papers that used this architectural paradigm have indeed achieved state-of the-art results [71,72] in many natural language processing (NLP) tasks. Dai et al. [57] were the first to employ a Transformer-based architecture for the MRI-based knee injury detection task. In particular, their hybrid (Transformer and CNN) model was used to extract features that pick up the long-range dependencies between MRI and other modalities.

The present review demonstrated that the prediction accuracy of the DL models for the ACL and meniscus tears detection ranged from 72.5% to 100%. However, certain limitations have been identified in all studies that are included in this literature review. The lack of multi-center data has been recognized as a limitation in three papers [15,27,53], leading to the development of biased DL-detection systems that have only been tested on knee MRIs carried out at a single institution. The results of these studies relate to knee examinations using specific MR acquisition protocols for knee joint assessment. In general, classification models, trained using data acquired by a specific MRI acquisition protocol, are unsuccessful or underperform when applied to data that was obtained differently. One way to tackle this lack of ability to generalize is by using DL models that learn MRI acquisition-invariant contrast-agnostic representations [73,74]. The effect of data imbalance has been also highlighted in some cases [6,54,55] where the sample of patients was not properly balanced among all gradings, leading the algorithms to pay more attention to the majority class (typically the class of healthy subjects). Applying down-sampling in the majority class has been proved to be an unreliable approach, which led to a biased result in the case of the fully ruptured classes [55]. Verification bias was also identified [24,53], mainly because subjects involved in the studies underwent arthroscopic knee surgery, leading to increased sensitivity and decreased specificity for both the detection system and the clinical radiologists. Moreover, it should be stressed that the grades used for the training of the detection algorithms are typically dependent on subjective assessment by a limited number of radiologists (one in some cases [54]). In most of the studies, only two categories (normal versus tears) were discriminated and the need of considering additional categories was highlighted [6] to allow more detailed classifications to happen. Overall, it was stressed that the diagnostic performance of the combined use of a clinical radiologist and machine interpretation of the MRI examinations has not been evaluated [53].

The current study is a systematic review that followed the PRISMA guidelines, but did not include a more formal quantitative meta-analysis due to the observed heterogeneity of the identified studies. Moreover, diagnostic arthroscopy was not used as the gold-standard reference to identify ACL or meniscus injuries in the majority of the studies, which may restrict the clinical applicability of the findings.

Future studies should try to train and test the accuracy of AI prediction models for the detection of ACL and meniscal lesion based on the arthroscopic images, and compare the outcome with that of direct, non-arthroscopic assessments. Arthroscopy is a surrogate “gold-standard” for the validation of non-invasive assessments, such as MRI, as it provides highly magnified and direct views of articular cartilage with non-destructive interactive assessments of its structure and functional properties.

Radiological imaging data of the knee continues to grow at a disproportionate rate, vastly outnumbering the trained MSK radiologists. The workload has also increased dramatically, leading to inevitable errors in the decision-making process. Despite the identified limitations, AI systems have the potential to relieve physician burnout, utilize clinicians in fields at which they have not been specialized (MSK MRI), and reduce the cost of knee injury diagnosis for the public health system. In addition to flagging abnormal cases, if an AI algorithm could rapidly identify negative exams (increased sensitivity and negative predictive value), then, a substantial amount of time and other resources could be made free. Such a concept would be really useful in countries without easy access to medical expertise.

Advances in medical imaging, in terms of quality, sensitivity, and resolution, have enabled the discrimination between the smallest differences in the various knee tissue densities. These differences sometimes are difficult to recognize, even by a trained, specialized eye. Expert’s diagnostic capacity used to be superior, but now we see this has been balanced out. As it was recently reported [24], deep CNN performance has reached performance levels akin to fellowship-trained, full-time, academic MSK radiologists in several tasks, including detection and segmentation. Despite this, AI can provide several new tools to the field of radiology imaging of the knee and medicine in general. The major hope for automated intelligent systems in the knee injury diagnosis is to increase accuracy, efficiency, and productivity in order to streamline patient care and outcomes. The newest, high-performance DL models should surpass the performance of traditional systems, meet the requirements for clinical utility, and become more user-friendly for the MSK clinician. Furthermore, there is the possibility of better training for young MSK radiologists with the help of AI.

MRI data of the knee, complemented by massive amounts of associated, multi-dimensional data such as omics and electronic health records, are only expected to grow. To fully exploit the full potential of this wealth of data, new paradigms should arise involving processes and workflows suitable for multi-institutional collaboration. Moreover, addressing the need for trustworthy detection systems of knee injuries, a medical diagnosis algorithm should meet a number of requirements (e.g., transparency, interpretability, explainability, and ease of use) in order to gain trust from clinicians. AI explainability and lightweight deep learning are key enablers for the wide use of such systems in the everyday clinical practice. Exploiting the intersection and merits of traditional ML and DL methods, AI analytics are expected to revolutionize knee medical informatics, enabling informed and accurate diagnoses needed by precision medicine.

Notwithstanding the huge potential of AI to improve the medical domain, the DL-based methods have yet to achieve significant deployment in clinical environments. This mainly ensues as a result of: (i) the intrinsic black-box nature of the DL algorithms; and (ii) the high computational cost. Explainable AI aims at building trust in the AI algorithms by providing medical experts with a diagnostic rationale behind the AI decision processes. The goal of the lightweight DL field is to develop models that have shallower architecture and are also faster and more data-efficient, while retaining the high-performance standards. Jeon et al. [56] were the first to get to grips with the clinical deployment of the MRI-based knee injury diagnosis. To this end, they proposed to use post-inference visualisation tools (such as CAM and Grad-CAM), and they also incorporated attention modules, Gaussian positional encoding, squeeze modules, and fewer convolutional filters.

## Figures and Tables

**Table 1:** Brief presentation of the feature extraction techniques, as well as the ML and DL models, and the main procedures that were reported in the papers of our survey.

Category	Models	Description
Feature extraction	Histogram of oriented gradient (HOG) [35]	This is a feature descriptor used in computer vision and image processing for the purpose of object detection. The technique counts occurrences of gradient orientation in localized portions of an image.
	Generalized search tree (GIST) [30]	GIST descriptor represents holistic spatial scene properties (spatial envelope) of an image. It summarizes gradient information on different spatial scales and orientations by splitting the image into a grid of cells on several scales and convolving each cell using a Gabor filter bank from different perspectives.
	Gray-level co-occurrence matrix (GLCM) [36]	GLCM is a way of extracting second-order statistical texture features. In particular, the texture of an image is estimated by calculating how often pairs of pixels with specific values and a certain spatial relationship occur.
Traditional Machine Learning	k-nearest neighbor (K-NN) [37]	KNN algorithm is a simple, easy-to-implement supervised ML algorithm that can be used to solve both classification and regression problems. It works by (i) finding the distances between a query and all the examples in the data, (ii) selecting the K nearest neighbors of the query, and (iii) voting for the most frequent label (in the case of classification) or averaging the labels (in the case of regression).
	Support vector machines (SVMs) [38]	SVMs is a supervised method that identifies a hyperplane that best divides the data into two classes. To separate the two clouds of data points, there are many possible hyperplanes that could be chosen. The objective of the SVM algorithm is to find a slab that has the maximum thickness, i.e., the maximum distance between data points of the different classes.
	Shallow artificial neural networks (ANNs) [39]	The ANN vaguely simulates the way the human brain analyzes and processes information. They consist of sequential layers: input, hidden and output layers. The hidden layer processes and transmits the input information to the output layer.
Deep Learning	Convolutional neural networks (CNNs) [40]	This is a class of DL algorithms commonly used in computer vision and pattern recognition. CNNs are a specific type of neural networks that are generally composed of the following layers: (i) input layer, (ii) convolution layers, (iii) pooling layers and (iv) fully connected layers. The convolution layers use filters that perform convolution operations as they are scanning the input with respect to its dimensions. Pooling is a down-sampling operation, which is typically applied after a convolution layer. The fully connected layers operate on a flattened input where each input is connected to all neurons in the next layer and are usually found towards the end of CNN architectures to optimize objectives such as class scores.
	Region based convolutional neural networks (R-CNNs) [41]	The method of detecting and classifying objects in an image is known as object detection. R-CNN (regions with convolutional neural networks) is a deep learning technique that blends rectangular area proposals with convolutional neural network functionality. The R-CNN algorithm is a two-stage detection method.
	Deep residual networks [42]	A residual neural network (ResNet) is an ANN variant that uses residual mapping and shortcut connections to tackle the problem of vanishing and exploding gradients that is characteristic of deep CNNs. As a consequence of this, deep residual networks achieve better performance when compared to plain very deep networks, whereas their training is easier as well. Typical ResNet models are implemented with double- or triple-layer skips that contain nonlinearities such as rectified linear unit (ReLUs) and batch normalization in between.
	3D-CNNs [43]	A 3D CNN is simply the 3D generalization of 2D CNNs. It takes as input a 3D volume or a sequence of 2D frames (e.g., slices in an MRI scan). Then kernels move through 3 dimensions of data producing 3D activation maps. Overall, they learn powerful representations of volumetric data.
	Computer Vision Transformers [44]	When data is modelized as a sequence of embeddings, the Transformer model is a basic yet scalable technique that can be used for any type of data. Even without typical convolutional pipelines, transformers can be utilized to provide SOTA results in Computer Vision. It is a DL network that extracts inherent properties of the interest domain via the self-attention technique.
Procedure	Training	The standard procedure involves a dataset of paired images and labels (x, y) for training and testing, an optimizer (e.g., stochastic gradient descent, Adam [45]), and a loss function to update the model parameters. The aim of the training is to find the optimal values for the network parameters so that the loss function is minimized.
	Data augmentation	Data augmentation is a strategy that artificially generates more training samples to increase the diversity of the training data. This can be done via applying affine transformations (e.g., rotation, scaling), flipping or cropping to original labeled samples.
	Dropout	Dropout is a regularization method that randomly drops some units from the neural network during training, encouraging the network to learn a sparse representation. It is used to reduce overfitting.
	Loss function	The metric to assess the discrepancy between model predictions and labels is called loss function. The gradients of the loss function are used to update the weights of the neural networks.
	Transfer learning	This aims to transfer knowledge from one task to another different but related target task. This is often achieved by reusing the weights of a pre-trained model, to initialize the weights in a new model for the target task. Transfer learning can help to decrease the training time and achieve lower generalization error.

**Table 2:** Results of studies.

No.	Author	Year	AI Model Used	Pretrained CNN	MRI (T)	Localization Technique	Validation	Performance (Accuracy/AUC)	Application Domain
1	Awan et al. [55]	2021	CNN	ResNet-14	1.5 T	They applied normal approach to localize based upon region of interest (ROI)	5-fold cross-validation	92%/(healthy tear = 0.98, partial tear = 0.97 and fully ruptured tear = 0.99)	ACL tear
2	Jeon et al. [56]	2021	3D CNN	VGGNet, AlexNet, and SqueezeNet	3 T & 1.5 T	Custom localization technique	5-fold cross-validation	N/A/0.983 and 0.980 on the Chiba and Stanford knee datasets, respectively	ACL tear
3	Rizk et al. [65]	2021	3D CNN	CNN-based localization model	1 T (54%)–1.5 T (9.7%)–3 T (36.3%)	Custom localization technique	ten-fold cross validation	Meidal = N/A/0.93, Lateral = N/A/0.84	Meniscus tear
4	Dai et al. [57]	2021	TransMed	N/A	3 T & 1.5 T	N/A	120 exams	ACL tear = 94.9%/0.98, Abnormality = 91.8%/0.976, Meniscus tear = 85.3%/0.95	ACL tear—Meniscus tear—Abnormalities
5	Astuto et al. [58]	2021	3D CNN	N/A	3 T	V-Net	Hold out (15% of sample)	N/A/from 0.83 to 0.93	ACL tear—Meniscus tear—Cartilage Lession
6	Fritz et al. [15]	2020	DCNN	N/A	1.5 T (64%)–3 T (36%)	To visually localize the tear, the software computes the class activation map (CAM) of the last convolution layer in the CNN and maps it to an axial knee image	Hold out (10% of sample)	Medial = (86%/0.88), Lateral = (84%/0.78), Overall = (N/A/0.96)	Meniscus tear
7	Namiri et al. [54]	2020	CNN	N/A	3 T	three-dimensional V-Net	Hold out (10% of sample)	3D-model = (89%/sensitivity of 89% and specificity of 88%), 2D-model = (92%/sensitivity of 93% and specificity of 90%)	ACL tear
8	Zhang et al. [6]	2020	CNN	3D DenseNet, VGG16, ResNet	1.5 T (74%)–3 T (26%)	-	Hold out (20% of sample)	Custom = (95.7%/0.96), ResNet = (NA/0.95), VGG16 = (NA/0.86)	ACL tear
9	Germann et al. [24]	2020	DCNN	N/A	1.5 T–3 T	They cropped manually	Out of the 5802 MRI studies, 4802 were used for training, 500 for validation, and 500 for initial testing	N/A/0.94	ACL tear
10	Azcona et al. [52]	2020	CNN	MRNet, ResNet18, Resnet50 and ResNet152, ImageNet	3 T (56.6%)–1.5 T (43.4%)	-	N/A	NA/0.96–N/A/0.91–N/A/0.94	ACL tear—Meniscus tear—Abnormalities
11	Chang et al. [8]	2019	CNN	ResNet	1.5 T–3 T	The object localization CNN was implemented as a fully convolutional network based on U-net architecture	5-fold-cross-validation	96.7%/0.97	ACL tear
12	Liu et al. [53]	2019	CNN	LeNet-5, DenseNet, VGG16, AlexNet	N/A	They used object detection technique YOLO	50 subjects test set (14% of the sample)	N/A/0.98	ACL tear
13	Couteaux et al. [61]	2019	CNN	ResNet-101, ConvNet, R-CNN	N/A	To localize both menisci and identify tears in each meniscus, they used the Mask R-CNN framework	54 cases and the model with the highest validation accuracy was selected	N/A/0.90	Meniscus tear
14	Pedoia et al. [63]	2019	2D U-Net, CNN	N/A	3 T	-	Hold out (20% of sample)	Sensitivity of 89.81% and specificity of 81.98%	Meniscus tear
15	Roblot et al. [62]	2019	CNN	AlexNet, MRNet	N/A	They used object detection technique Fast RCNN & Faster RCNN	The algorithm was thus used on a test dataset composed of 700 images for external validation	72.5%/0.85	Meniscus tear
16	Nicholas Bien et al. [27]	2018	CNN	AlexNET, MRNet	3 T (56.6%)–1.5 T (43.4%)	-	120 exams	86.7%/0.97–72.5%/0.85–N/A/0.94	ACL tear—Meniscus tear—Abnormalities
17	Liu et al. [66]	2018	CNN	VGG16	3 T	-	fellowship trained musculoskeletal radiologist (R.K., with 15 years of clinical experience)	N/A/0.92	Cartilage lesion
18	Stajduhar et al. [48]	2017	HOG + linSVM, HOG + RF, GIST + rbfSVM, GIST + RF	N/A	1.5 T	Manual extraction of a rectangular ROI	10-fold cross validation	(Injury detection problem, complete rupture) = (N/A/0.89, N/A/0.94), (N/A/0.88, N/A/0.94), (N/A/0.889, N/A/0.91), (N/A/0.88, N/A/0.90) respectively with the models	ACL tear
19	Mazlan et al. [51]	2017	SVM	N/A	N/A	They use cropping technique	Hold out (10% of sample)	100%/N/A	ACL tear
20	Zarandi et al. [60]	2016	IT2FCM, PNN	N/A	N/A	-	Hold out (20% of sample)	0 and 1 mode: 90%/N/A Binary mode: 78%/N/A	Meniscus tear
21	Fu et al. [59]	2013	SVM	N/A	N/A	Active Contours without Edges method. This method combines Active Contours with Level Sets and is called ACLS	5-Fold cross validation	SVM model: N/A/0.73 SFFS + SVM: N/A/0.91	Meniscus tear
22	Abdullah et al. [50]	2013	BP ANN, K-NN	N/A	N/A	-	5-fold and 6-fold	BP ANN: 94.44%/N/A k-NN: 87.83%/N/A	ACL tear

## Supplementary Materials

The following supporting information can be downloaded at: https://www.mdpi.com/article/10.3390/diagnostics12020537/s1.
