# Supplementary material for: Knee Injury Detection Using Deep Learning on MRI Studies: A Systematic Review
Source: Diagnostics (Basel). 2022 Feb 19;12(2):537. doi: 10.3390/diagnostics12020537 (PMC8871256; doi:10.3390/diagnostics12020537)
Supplement: Supplementary file 1 [file diagnostics-12-00537-s001.zip › diagnostics-1546933-supplementary.pdf]

diagnostics

Table S1. Terms for strategy search.

Database	Terms
**MEDLINE:**	((((((((deep learning) OR (cnn)) OR (convolutional neural networks)) OR (machine learning))) OR (artificial intelligence)) OR (neural networks)) AND (((((knee injury) OR (ACL tear)) OR (Anterior crucial ligament)) OR (knee menisc*)) OR (knee cartilage))) AND (image)
**CENTRAL:**	((((((″ deep learning″) OR (″ machine learning″)) OR (″ neural networks″)) OR (″ CNN″)) AND (knee)) AND (image)) NOT (osteoarthritis)- in All Text— (Word variations have been searched)
**EMBASE:**	find= (deep learning OR neural networks OR machine learning) AND Title= (knee AND image NOT osteoarthritis)
